# Impact of a multidisciplinary intervention on Mediterranean diet adherence and cardiovascular health

**DOI:** 10.1093/eurpub/ckac131.342

**Published:** 2022-10-25

**Authors:** E Marziali, S De Marco, L Nachira, P Arcaro, F D'Ambrosio, L Villani, V Galasso, P Laurenti, S Bruno

**Affiliations:** 1 Department of Life Sciences and Public Health, Università Cattolica del Sacro Cuore, Rome, Italy; 2 Università degli Studi di Roma Tor Vergata, Rome, Italy; 3 DiagnostiCare ONLUS, Rome, Italy; 4 Department of Woman, Child Health and Public Health, Fondazione Policlinico Universitario A. Gemelli IRCCS, Rome, Italy

## Abstract

**Background:**

Cardiovascular diseases represent a significant public health issue, and the promotion of healthy lifestyles plays a major role in their prevention. Good adherence to the Mediterranean diet has a protective effect on cardiovascular health and may reduce the risk of developing cardiovascular disease. Our prospective study aimed to evaluate the impact of educational lifestyle interventions on cardiovascular risk parameters and the improvement in adherence to the Mediterranean diet of the involved population.

**Methods:**

Participants have been recruited by General Practitioners in Torresina neighborhood in Rome. From December 2018 to June 2020, 41 patients were involved in nutritional, psychological, and physical activity meetings by a multidisciplinary team of healthcare professionals. In particular, a nutritionist provided information to patients on balanced nutrition, considering the Mediterranean diet as a dietary model. Information on lifestyle, dietary habits and physical activity, anthropometric data and laboratory measurements were collected at baseline and after 12 months. The variations of the evaluated parameters were analyzed by paired t-test e Wilcoxon signed-rank test.

**Results:**

The analysis showed statistically significant decreases in weight (p = 0.03) and BMI (p = 0.02), as well as in systolic (p < 0.001) and diastolic (p = 0.001) blood pressure and in total (p = 0.02) and LDL (p = 0.01) cholesterol level. Results also showed an improvement in the adherence to the Mediterranean diet (p = 0.001): the frequency of consumption of fruits and vegetables, legumes, cereals and fish has increased significantly, while the consumption of meat, milk and dairy products and alcohol decreased.

**Conclusions:**

This study highlights that a multidisciplinary educational program can be effective in improving healthy habits and in reducing cardiovascular risk factors, supporting its implementation in primary prevention at the community level.

**Key messages:**

• Promoting healthy lifestyle through primary prevention and health promotion actions is critical to reduce the onset of cardiovascular diseases.

• A population-based multidisciplinary educational intervention may be effective in improving adherence to a healthy, balanced diet and decreasing cardiovascular risk factors.

